# Real-time execution of SNN models with synaptic plasticity for handwritten digit recognition on SIMD hardware

**DOI:** 10.3389/fnins.2024.1425861

**Published:** 2024-08-06

**Authors:** Bernardo Vallejo-Mancero, Jordi Madrenas, Mireya Zapata

**Affiliations:** ^1^Department of Electronic Engineering, Universitat Politècnica de Catalunya, Barcelona, Spain; ^2^Centro de Investigación en Mecatrónica y Sistemas Interactivos—MIST, Universidad Indoamérica, Quito, Ecuador

**Keywords:** HEENS, neuromorphic hardware, spiking neural network, LIF model, Spike Timing Dependent Plasticity, MNIST dataset, homeostasis, FPGA

## Abstract

Recent advancements in neuromorphic computing have led to the development of hardware architectures inspired by Spiking Neural Networks (SNNs) to emulate the efficiency and parallel processing capabilities of the human brain. This work focuses on testing the HEENS architecture, specifically designed for high parallel processing and biological realism in SNN emulation, implemented on a ZYNQ family FPGA. The study applies this architecture to the classification of digits using the well-known MNIST database. The image resolutions were adjusted to match HEENS' processing capacity. Results were compared with existing work, demonstrating HEENS' performance comparable to other solutions. This study highlights the importance of balancing accuracy and efficiency in the execution of applications. HEENS offers a flexible solution for SNN emulation, allowing for the implementation of programmable neural and synaptic models. It encourages the exploration of novel algorithms and network architectures, providing an alternative for real-time processing with efficient energy consumption.

## 1 Introduction

In recent years, the field of neuromorphic computing has witnessed significant advancements, driven by the quest to emulate the remarkable efficiency and parallel processing capabilities of the human brain. Opposite to mainstream Artificial Neural Networks (ANNs), Spiking Neural Networks (SNNs) provide a biologically close framework for information processing (Maass, 1997), attempting to integrate more biological mechanisms (Schmidgall et al., 2023). SNNs communicate through spikes, a strategy that mimics the neural activity observed in biological neurons. This spike-based communication enables SNNs to exhibit temporal dynamics and local adaptation, characteristics that closely resemble those found in biological neural networks (Malcolm and Casco-Rodriguez, 2023). This approach aims to simplify the power- and resource-consuming training demanded by ANNs because of two factors. Based on events, SNNs can theoretically execute with reduced power consumption compared to a continuous execution. Furthermore, the local plasticity algorithms, which refer to the ability of a neural network to adapt and change its structure or parameters in response to new information or experiences (Jordan et al., 2021), reduce the computation load compared to global gradient-descent methods. Despite living beings demonstrate that it is possible to achieve excellent results in recognition and classification tasks, ANNs are today still superior to SNNs in most of those tasks. Therefore, more research is needed in unraveling biological keys and proposing SNN topologies and algorithms (Diehl and Cook, 2015; Yang et al., 2024b).

In order to improve the knowledge and understanding of SNNs, the availability of specific hardware allows their fast prototyping and real-time execution. In order to make progress in demonstrating their suitability for solving real-world problems, it is necessary to map applications and benchmark them. Two possible reasons may explain why the results generally do not achieve the same performance as classic ANNs. First, the attempt to reproduce the same topology and configuration of ANNs by encoding values on spike streams may lead to sub-optimal solutions. Second, the development of efficient local adaptation algorithms is still an open research field, where advancements continue to be made in enhancing robustness and energy efficiency through innovative learning techniques (Yang and Chen, 2023; Yang et al., 2023).

So far, many neural and synaptic plasticity models have been proposed (Sanaullah et al., 2023), but often it remains unclear what is the best usage of them and the most suitable spike encoding. However, the fact that biological networks are able to perform excellent recognition tasks is a motivation to search and a hint that good performance can be achieved with current SNNs models or their evolutions.

In order to accelerate the production of results and reduce the resources demanded by large-scale networks, a promising avenue in this domain is the development of custom hardware architectures that combine low power consumption and high computational efficiency with real-time execution. For proof of concept demonstration, architectures implemented on Field Programmable Gate Arrays (FPGAs), excel in fast prototyping (Akbarzadeh-Sherbaf et al., 2018; He et al., 2022; Yang et al., 2024a), at the cost of resource count and power dissipation compared to ASICs.

Application-Specific Integrated Circuits (ASICs) allow to obtain the maximum performance from a given chip technology, at a much higher development cost. Among the most significant proposed ASIC architectures supporting SNNs, the following stand out: IBM TrueNorth (DeBole et al., 2019), a real-time neurosynaptic processor that features a non-von Neumann, low-power, highly-parallel architecture, with a new version called PoleNorth (Cassidy et al., 2024) ; SpiNNaker (Mayr et al., 2019), composed of multiple ARM processors that support a general-purpose architecture with multiple instructions and multiple data; and finally, the Intel Loihi 2 chip (Orchard et al., 2021), one of the most advanced chips with asynchronous operation. As an important added asset, SNN-specific hardware also offers the possibility of interfacing with sensors to interact with algorithms in real time using the data produced.

Contributing to the neuromorphic field, the Hardware Emulator of Evolving Neural Systems (HEENS) is being developed as a digital synchronous architecture designed to emulate SNN with high levels of parallel processing, reduced resource and power requirements, keeping the synchronous digital flexibility, and focusing on real-time neural emulation with biological realism and user-friendly prototyping environment.

The development of HEENS is based on significant previous research. For instance, originally Madrenas and Moreno (2009) introduced a scalable multiprocessor architecture employing SIMD configurations that is flexible for emulating various neural models. Additionally, the implementation of the AER communication protocol, detailed by Moreno et al. (2009) and Zapata (2016), has been crucial in enabling compact emulation of interconnections in large-scale neural network models. Also notable is the Spiking Neural Networks for Versatile Applications (SNAVA) simulation platform (Sripad et al., 2018), a scalable and programmable parallel architecture implemented on modern FPGA devices. The integration of these technologies and other advances implemented over time have allowed the current development of HEENS, which incorporates features of these previous works while introducing new capabilities that are described in the following sections.

This article provides a brief overview of HEENS, delving into its architectural characteristics features and focusing on its remarkable flexibility in prototyping SNNs, designed to adapt and emulate a wide variety of neural configurations, and its programmability, allowing researchers and developers to customize and adjust the emulator's behavior according to the specifications of their neural applications. In this context, the central objective of this work is to present the detailed methodology for modeling and implementing applications of SNN. The work starts with an exploration of its fundamental components, described in Section 2.1 namely a summary of the HEENS hardware architecture, followed by an in-depth analysis of neural and synaptic models, including the Leaky Integrate and Fire (LIF) neuron model and Spike Timing Dependent Plasticity (STDP) mechanisms (Bliss and Gardner-Medwin, 1973). In addition, we will assess the concept of homeostasis (Ding et al., 2023) and its impact on network behavior. These concepts will be demonstrated by implementing a handwritten digit recognition application in HEENS, using data sets such as MNIST (LeCun et al., 1998) to train and test neural networks. Through a series of experiments, we evaluate HEENS' effectiveness in achieving precision and robustness in pattern recognition tasks, comparing its performance with alternative solutions and identifying potential limitations.

This article aims to provide valuable insights into the capabilities and potential of HEENS by means of the demonstration of a character recognition application with local learning to advance the frontier of neuromorphic computing for researchers and professionals. With its blend of biological realism, scalability, and computational efficiency, HEENS emerges as a promising platform for exploring the intricacies of neural information processing and accelerating the development of intelligent systems.

Following this introduction, in Section 2, the methodology used is detailed, including architectural description, network architecture, neural and synaptic modeling, culminating in the implementation of the neural application, as well as the proposed input encoding and the stages of training and evaluation. Section 3 presents the execution experiments and results, while Section 4 offers a discussion of the results and the comparison with other alternatives or solutions, ending with future work and directions. Finally, the conclusion is reported in Section 5.

## 2 Methods

### 2.1 Summary of the HEENS hardware architecture

**Figure 1:**
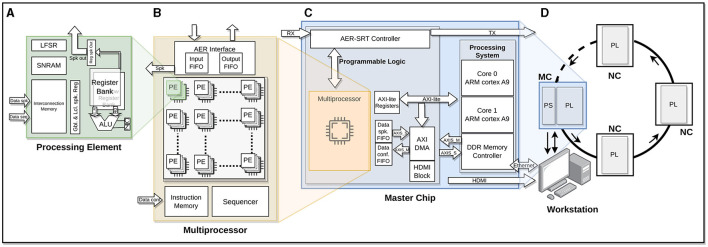
HEENS architecture overview. **(A)** Processing Element (PE) scheme. **(B)** Multiprocessor. **(C)** Master chip: Processing System (PS) and Programmable Logic (PL). **(D)** Ring topology in a master-slave connection scheme: master chip/node (MC) and neuromorphic chips/nodes (NC).

HEENS is a Single Instruction Multiple Data (SIMD), scalable, and multichip hardware architecture implemented on several devices of the Xilinx's Zynq family of Field Programmable Gate Arrays (FPGAs), designed to emulate multi-model SNNs with high parallelism (Figure 1), using basic Processing Elements (PE) adapted to SNN requirements. PE arrays allow for the implementation of clustered networks inspired by the structural organization of the brain. The main goal of HEENS is to emulate large-scale SNNs in real time with a high degree of biological realism while also providing users with a friendly and flexible prototyping environment.

The basic system unit (PE, Figure 1A) contains the necessary logic to emulate one or more biological neurons and their synapses. It is composed of the fundamental units of a processor datapath: an Arithmetic and Logic Unit (ALU), a register bank, and routing resources, as well as two local Static Random Access (SRAMs). Each PE's ALU performs hardware addition, subtraction, multiplication, and logical operations, interacting with a 16-bit general-purpose register bank and an additional shadow register bank for extended register space. The Interconnection Memory stores the synaptic connection map. It is used to decode the incoming spike address identifier, detecting those that match the PE, while the Synaptic and Neural RAM (SNRAM) contains the neuron parameters required for the execution of the synaptic and neural models. A 64-bit Linear Feedback Shift Register (LFSR) is also included in each PE to generate uncorrelated noise, essential for various neural applications.

Each PE can be time-multiplexed without the need for additional hardware, at the cost of increasing execution time. This process is referred to as virtualization, which allows emulating (i.e., executing in real time) a maximum of eight virtual neurons per PE.

A 2D PE array together with the control unit, consisting of a Sequencer and an Instruction Memory, form the multiprocessor (Figure 1B). HEENS has its own custom Instruction Set Architecture (ISA), especially designed for the implementation of arbitrary spiking neural and synaptic models. The neural and synaptic programs are stored in the Instruction Memory. The sequencer performs instruction fetch and decoding, executes the control instructions, and broadcasts the ones related with data processing to the PE array. Finally, a synchronous Address Event Representation (AER) interface is in charge of internally and externally convey the spikes produced by the PE array neurons.

The architecture supports a maximum array of 16 × 16 PEs, which allows emulating up to 16 × 16 × 8 (2k) neurons with 256 synapses per PE distributed among its virtual layers. However, the total number of PEs that can be implemented depends on the hardware resources available in the FPGA. In the case of the Zynq706 (the model used in this work), an array of 16 × 10 PEs has been successfully mapped operating at a frequency of 125 MHz.

In Figure 1C, the main blocks that make up a master chip (node) can be observed. The master node includes communication with the user. This node consists of two parts, associated with the Programmable Logic (PL) and the Processing System (PS) of the programmable device. The PL contains the multiprocessor along with dedicated communication blocks for user interaction and the transmission of local and global spikes. On the other hand, the PS, which in the case of Zynq family chips embeds two ARM family processors, acts as a bridge between the user and the multiprocessor implemented in the PL, allowing the transmission of configuration packets and network control, monitoring and debugging information.

To support working with more complex networks and a larger number of neurons, the architecture can be extended by connecting slave nodes, creating a master-slave ring topology (Figure 1D), where a Master Chip is used along with Neuromorphic Chips (NC) as slaves. The latter contain multiprocessor instances, but lack the PS. The addressing space of the architecture supports up to 127 NCs. The communication between the chips is done using the Address Event Representation over Synchronous Serial Ring Topology (AER-SRT) protocol due to its high performance in applications focused on event transmission through GTX high-speed serial transceivers (Dorta et al., 2016). This communication is used not only to transmit spikes, but also to send configuration packets to each of the chips. Besides the monitoring through the master node, it is also possible for any node to support real-time monitoring via HDMI (Vallejo-Mancero et al., 2022).

Among the main features of HEENS, the following can be highlighted.

- Real-time operation. Time slots of 1 ms are considered real-time for each execution cycle of neural applications. This time slot can be tuned.
- High scalability: Achieved through an extendable and compact communication system, and low-resource, low-power design. Notice that each node requires only an input and an output serial port for all the operations.
- Unified data flow: The same communication ring supports all tasks: System configuration, spike transmission, and system execution monitoring.
- Multimodel Algorithm Support: Virtually any spiking neural algorithm can be programmed, including LIF (Abbott, 1999), Izhikevich (Izhikevich, 2003) , Quadratic LIF (Alvarez-Lacalle and Moses, 2009), and others, using dedicated software tools.
- Flexible Synaptic Algorithms: Same as neural ones, local synaptic algorithms are customizable via software, including Spike-Timing Dependent Plasticity (STDP) (Bliss and Gardner-Medwin, 1973), as a relevant example.
- Efficient Memory Usage: The neural, synaptic, and connection parameters are stored in local memory for optimized performance. This solves the processor-memory bottleneck issue by applying the *in-memory* computing paradigm.
- Comprehensive Computer-Aided Engineering (CAE) Toolset: Tools that fully automate the configuration and real-time monitoring of SNNs, making the mapping of a neural network a simple task of specifying in textfiles the network topology, the initial neural and synaptic parameters and selecting from a library the neural and synaptic algorithm to be executed (Oltra et al., 2021; Zapata et al., 2021).

#### 2.1.1 External input module

**Figure 2:**
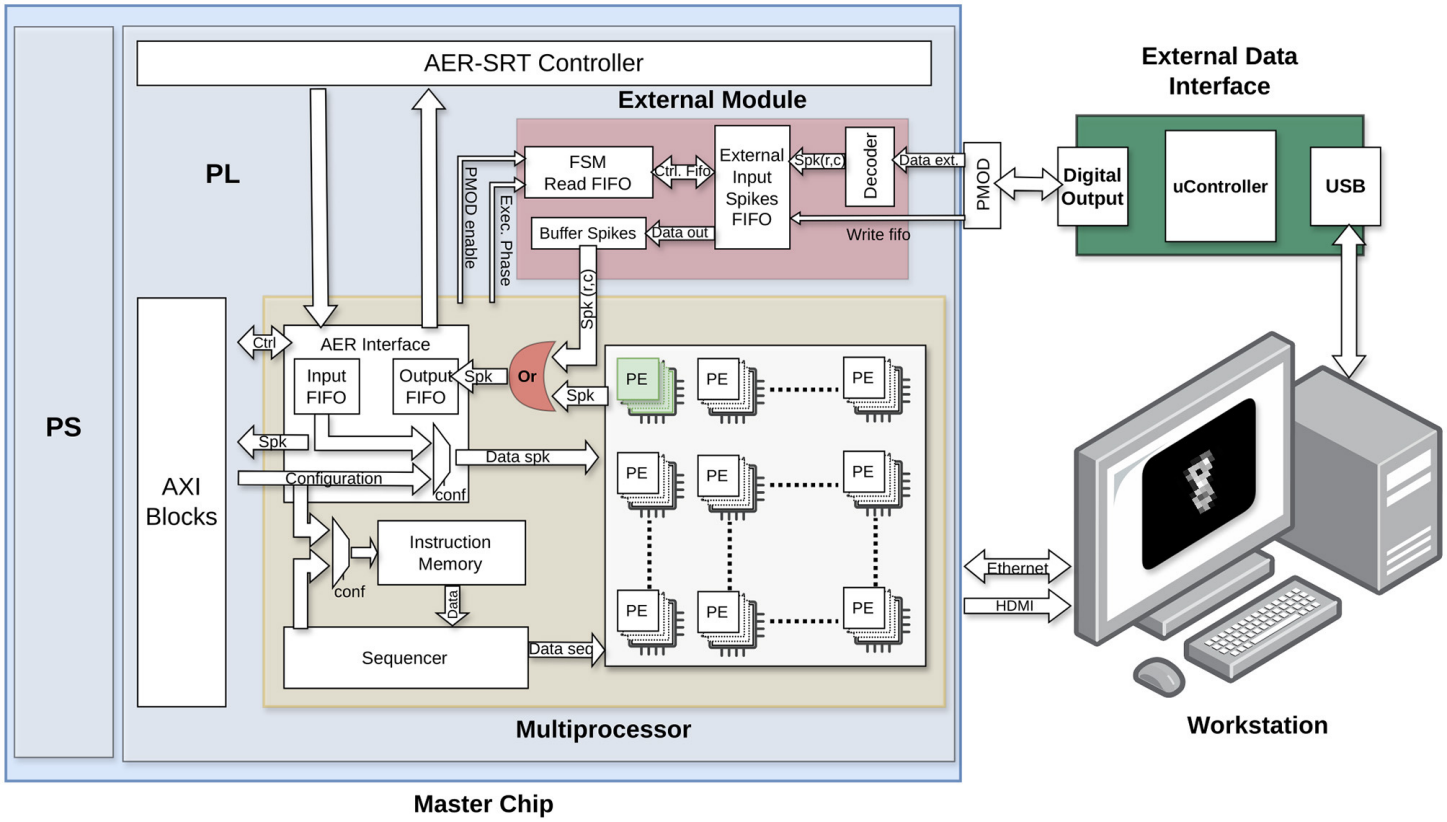
Schematic of the external spike input module hardware, detailing its individual blocks and connection with other modules.

As observed in Figure 2, the original HEENS architecture was adapted to directly support access to the PE by multiplexing the output signal of the neuron. The PE array organization makes straightforward the direct access of external data to individual neurons. By default, the first virtual layer is accessed, although other layers could be equally accessed. This adaptation allows for the definition of a group of neurons that can be activated externally, managed by a hardware block responsible for converting the received signals into spikes directly injected to those specific neurons.

The conversion and injection of spikes into the system must consider the real-time processing performed by the architecture. To achieve this, a First-Input First-Output (FIFO) memory is utilized to store data until the corresponding execution cycle requires it, every time step (typically 1 ms). Data is transmitted from an external hardware block and, for the sake of increasing speed, each 8-bit data is encoded into two 4-bit packs transmitted in parallel and processed in the architecture by a decoder block. Each bit is directly connected to a destination register bit, reducing the number of clock cycles required for each data and allowing for faster data transmission to ensure the real-time operation.

Data can come from various sources, typically attached sensors, but for this work the MNIST dataset (LeCun et al., 1998) is utilized and externally generated, where each event has been encoded in an 8-bit word. An explanation of the dataset management is provided in Section 2.3.

### 2.2 Spiking neural and synaptic model

#### 2.2.1 SNN model

As mentioned before, arbitrary spiking neural models can be implemented in HEENS. The choice depends mainly on two factors: the degree of biological realism and the computational efficiency for the application. Considering the second factor, the LIF model (Abbott, 1999) has been selected. This model is recognized for its simplicity and low computational cost. This well-known model is described with Equations 1, 2.


(1) $$\begin{array}{l} \tau_{m}\frac{dV}{dt}=-\left(V\left(t\right)-V_{res}\right)+R\cdot I\left(t\right) \end{array}$$

(2) $$\begin{array}{l} \text{If: }V\left(t\right)\geq V_{th}\;\text{then:}V\left(t\right)\leftarrow V_{res} \end{array}$$

Where *V*(*t*) is the membrane potential, *V*_*rest*_ is the resting membrane potential, τ_*m*_ is the membrane time constant, *R* is the membrane resistance, *I*(*t*) is the synaptic input current and *V*_*th*_ is the threshold voltage. When the membrane potential crosses this threshold value, the neuron fires, and the membrane potential is reset to *V*_*rest*_. The calculation of the synaptic input current is represented by Equations 3–5.


(3) $$\begin{array}{l} I\left(t\right)=k.g_{ex}\left(t\right)+k.g_{ih}\left(t\right) \end{array}$$

(4) $$\begin{array}{l} \tau_{ex}\frac{dg_{ex}}{dt}=-g_{ex},\;\tau_{ih}\frac{dg_{ih}}{dt}=-g_{ih} \end{array}$$

(5) $$\begin{array}{l} g_{ex}=\overset{N_{ex}}{\underset{i=1}{\sum }}w_{i}\cdot \delta \left(t-t_{i}\right),\;g_{ih}=\overset{N_{ih}}{\underset{i=1}{\sum }}w_{i}\cdot \delta \left(t-t_{i}\right) \end{array}$$

Where *I*(*t*) represents the total input current received by the neuron at time *t*, which is computed as the sum of excitatory and inhibitory synaptic currents. The constant *k* is the synaptic potential difference, scaling the impact of excitatory and inhibitory synaptic conductances on the membrane potential of the neuron. The excitatory synaptic conductance dynamics, *g*_*ex*_(*t*), decays exponentially with τ_*ex*_ time constant, while *g*_*ih*_(*t*) represents the inhibitory synaptic conductance dynamics, following a similar exponential decay with τ_*ih*_ time constant. The weights *w*_*i*_ denote the strength of individual synapses, where *N*_*ex*_ and *N*_*ih*_ representing the total number of excitatory and inhibitory synapses, respectively. Finally, *t*_*i*_ represents the activation time of each synapse, indicating when a synaptic event occurs. These variables describe the complex dynamics of synaptic inputs and their collective influence on the neuron's total input current.

#### 2.2.2 Plasticity mechanism

In artificial neural networks (ANNs), learning techniques typically employ static algorithms to adjust connection weights based on specific training data, without dynamically incorporating the timing observed in biological neural network synapses (Abdolrasol et al., 2021). In contrast, synaptic plasticity mechanisms are dynamic processes through which connections between neurons can modify their strength and efficacy in response to neuronal activity, crucial for neural system functionality, particularly in learning and memory processes (Citri and Malenka, 2008). Several mechanisms contribute to synaptic plasticity dynamics, including Long-Term Potentiation (LTP), which strengthens synapses through repeated activation of presynaptic neurons, and Long-Term Depression (LTD), which weakens synapses during periods of reduced synaptic activity (Barco et al., 2008). Another form is Spike-Timing-Dependent Plasticity (STDP), which adjusts synaptic strength based on the precise timing of spikes between presynaptic and postsynaptic neurons.

The plasticity mechanism implemented in this work is STDP, described by Equation 6. It is a hardware-friendly version of the original Bliss and Gardner-Medwin (1973), that uses two new variables, *a*_*pre*_ and *a*_*post*_, known as pre and postsynaptic activity “traces” (Stimberg et al., 2019). These “traces” track the temporal activity patterns of presynaptic and postsynaptic neurons, respectively, where τ_*pre*_ and τ_*post*_ are their time constants that determine their rate at which these traces change over time. Due to the fact that the HEENS architecture processes spikes individually, it is important to highlight that the traces apply to individual spikes, not to spike rate averages.


(6) $$\begin{array}{l} \tau_{pre}\frac{da_{pre}}{dt}=-a_{pre},\;\tau_{post}\frac{da_{post}}{dt}=-a_{post} \end{array}$$

The weight or strength of the connection, denoted by *w*, adjusts dynamically with each presynaptic or postsynaptic spike, as shown in Equations 7, 8, in response to the temporal activity of the neurons. The Equation 9 ensure that the synaptic weight *w* remains within a biologically plausible range, without exceeding a maximum value *w*_*max*_ and without becoming negative.


(7) $$\begin{array}{l} a_{pre}\leftarrow a_{pre}+\text{Δ}a_{pre},\;w\leftarrow w+a_{post} \end{array}$$

(8) $$\begin{array}{l} a_{post}\leftarrow a_{post}+\text{Δ}a_{post},\;w\leftarrow w+a_{pre} \end{array}$$

(9) $$\begin{array}{l} w\leftarrow \{\begin{array}{cc} w_{max} & \text{if }w\geq w_{max},\\ 0 & \text{if }w\leq 0. \end{array} \end{array}$$

Where Δ*a*_*pre*_ and Δ*a*_*post*_ represent the increment and decrement amounts for synaptic plasticity variables, respectively.

Considering that a competitive learning method is employed, with the aim of each neuron or group of neurons to specialize in recognizing specific features, it is important to introduce a method to control and restrict the unlimited growth of weights between neurons, as mentioned in the work of Goodhill and Barrow (1994). To achieve this, a rule of synaptic scaling plasticity based on subtraction was implemented to generate competition among synapses. This mechanism normalizes synaptic strength after each training digit and follows Equations 10, 11.


(10) $$\begin{array}{l} fac_{w}=\frac{W_{norm}-\overset{N_{stdp}}{\underset{i=1}{\sum }}w_{i}}{N_{stdp}} \end{array}$$

(11) $$\begin{array}{l} w_{i}\leftarrow w_{i}+fac_{w} \end{array}$$

Where *fac*_*w*_ is the normalization factor of synaptic weight, *W*_*norm*_ represents the target value of normalization, *w*_*i*_ indicates the synaptic weight of the *i*-th synapse and *N*_*stdp*_ represents the total number of synapses subject to the STDP plasticity rule. These normalization techniques are already present in various image processing works, such as Krizhevsky et al. (2012).

#### 2.2.3 Homeostasis

The homeostasis involves biological systems maintaining stability by autonomously adapting to fluctuations in external conditions (Billman, 2020). In the context of neural networks, homeostasis ensures that neurons maintain balanced firing rates and receptive fields, preventing individual neurons from dominating neural responses. The homeostasis mechanism used here follows the same approach as Diehl and Cook (2015). The goal is to ensure that all neurons have similar firing rates but different receptive fields. To prevent a single neuron in the excitatory layer from dominating the response, the firing threshold (*V*_*th*_) was adapted in Equations 12, 13:


(12) $$\begin{array}{l} V_{th-ad}=V_{th}+\theta e^{-t/\tau_{hom}} \end{array}$$

(13) $$\begin{array}{l} If\;:V\left(t\right)\geq V_{th-ad}\text{ then}:\;\theta \leftarrow \theta +\text{Δ}\theta \end{array}$$

In the homeostasis equation, *V*_*th*−*ad*_ represents the adaptive threshold, *V*_*th*_ denotes the original threshold, and θ indicates the magnitude of the adaptation, which increases Δθ with each spike and decays exponentially according to the time constant τ_*hom*_. Consequently, as a neuron fires more frequently, its threshold progressively rises, needing greater input for subsequent firing. This iterative process persists until θ decreases to a satisfactory level.

### 2.3 Handwritten digit recognition

#### 2.3.1 MNIST dataset

The set of images used for the application corresponds to the MNIST dataset, which consists of grayscale images of handwritten digits from 0 to 9. It contains 60,000 training examples and 10,000 test examples. MNIST is widely used in the field of Optical Character Recognition (OCR), where applications for recognizing, various alphabets and languages such as the Latin alphabet (Singh and Amin, 1999; Darapaneni et al., 2020), Farsi-Arabic (Mozaffari and Bahar, 2012; Tavoli et al., 2018), Japanese (Matsumoto et al., 2001), besides numeric digits, are also notable. The MNIST dataset was chosen not only for its simplicity and clarity in digit recognition tasks but also because it serves as a standard benchmark dataset in the field of machine learning and neuromorphic computing (Guo et al., 2022; Liu et al., 2022; Tao et al., 2023). This selection allows for a direct comparison of the performance of the HEENS architecture with existing methodologies and provides a baseline for evaluating its capabilities.

**Figure 3:**
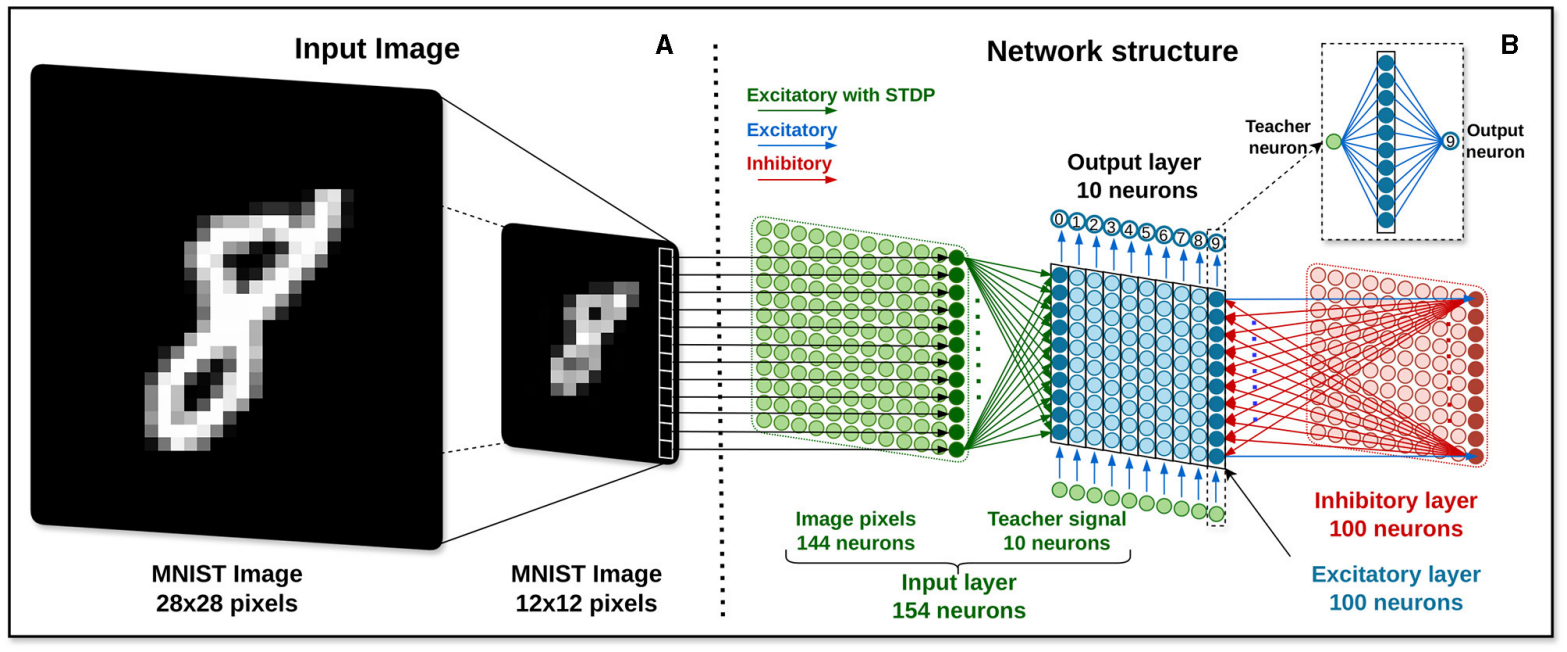
Image processing. **(A)** Input image encoding. **(B)** Network architecture.

Although each image has a size of 28 × 28 pixels, due to limitations regarding the maximum number of synapses and implementable neurons in the HEENS architecture, as explained in Section 2.1, the size of the images is reduced using the .resize((height, width)) function from the PIL (Python Imaging Library). This function resizes the image to the specified values assigned to the height and width variables using the nearest-neighbor interpolation technique (GeeksforGeeks, 2022). For this application, considering that the maximum number of neurons that can be implemented per node in HEENS is 16 × 10, the dimensions of each image are configured to be 12 × 12 pixels, resulting in a total of 144 pixels per input image. Each pixel corresponds to a neuron in the input layer, as illustrated in Figure 3A.

#### 2.3.2 Input encoding

The network receives input for 350 ms in the form of Poisson-distributed spike trains, with firing rates corresponding to the pixel intensity of MNIST images. Here, the maximum intensity, denoted as 255 for white and 0 for black, enables an accurate representation of luminosity levels within the images. Moreover, a 150 ms pause separates each data set digit presentation presentation, aiding in the clear differentiation of inputs.

In the training process, a supervised learning approach is adopted. A 200 Hz constant spike rate transmits the teacher signal, synchronized with the spikes generated by input images. This signal is conveyed through a designated input neuron, chosen based on the current class label being trained. This strategy is activated exclusively during the learning phase and is deactivated during testing. It facilitates the gradual enhancement of the network's performance in classifying various image classes, even in the absence of explicit error correction. Furthermore, it is worth noting that the teaching signal follows the same cycle duration of 350 ms with a 150 ms pause as the input presentation cycle.

### 2.4 Network architecture

The proposed network architecture consists of 4 layers, as illustrated in Figure 3B: the input layer, the excitatory neuron layer, the inhibitory neuron layer, and the output layer. It takes as reference the network architectures from Diehl and Cook (2015), Hao et al. (2020), and Lee and Sim (2023), with some variations.

Each pixel in the input image is connected one-to-one to 144 neurons of the input layer (in green color). These neurons are fully connected to the excitatory layer, being all connections excitatory with a synaptic plasticity mechanism enabled during the learning stage. The main function of these 144 neurons is to transmit encoded pulse trains from each image to the next layer.

In addition, the input layer contains 10 neurons (in green) driven by external input, used as teacher signals for supervised learning. Each of these neurons is excitatorily connected to a unique 10-neuron group of the excitatory layer. These reference signals have no configured plasticity mechanism and serve only to provide external information to guide the learning of the network without adjusting their own synaptic weights. The value of these connections is the maximum STDP weight defined in the constant *w*_*max*_.

The excitatory layer (in blue) consists of 100 LIF-type neurons with homeostasis enabled during the learning phase. In addition to the mentioned connections from the input layer, it also interacts with the inhibitory layer and the output layer. Each excitatory neuron establishes a direct excitatory connection with a corresponding inhibitory neuron characterized by a strong connection strength. In contrast, each inhibitory neuron forms inhibitory connections with all excitatory neurons in the layer, except the one to which it is directly connected. This setup aims to utilize the “winner take all” technique, where the first firing neuron inhibits the rest, aiding in classifying and selectively focusing the network toward the desired input. In the output layer, each column of 10 neurons is connected to an output neuron associated with a specific class (see Figure 3B inset). Thus, the first neurons are associated with *0*, the following ones with *1*, and so on up to *9*. The weight value used is 8 times *w*_*max*_, a value utilized in Hao et al. (2020) to increase the activity of the layer upon receiving spikes.

The inhibitory layer (in red), which consists of 100 LIF-type neurons with disabled homeostasis, plays a crucial role in regulating the activity of neurons within the excitatory layer. Each neuron within this layer receives input from an excitatory neuron. The synaptic weights of these connections must be carefully adjusted to prompt the activation of inhibitory neurons quickly upon receiving an excitatory pulse, ensuring timely inhibition and contributing to the network's ability to regulate and control its activity dynamics. At the same time, the weights of the inhibitory connections must be adjusted to achieve a balance that prevents the complete suppression of the participation of neighboring neurons in the computation of the network.

Finally, the output layer consists of 10 LIF neurons with disabled both homeostasis and STDP. This layer serves as the final processing stage in the neural network, with each neuron dedicated to representing one of the ten possible classified digits.

### 2.5 Neural and synaptic model implementation on HEENS

**Figure 4:**
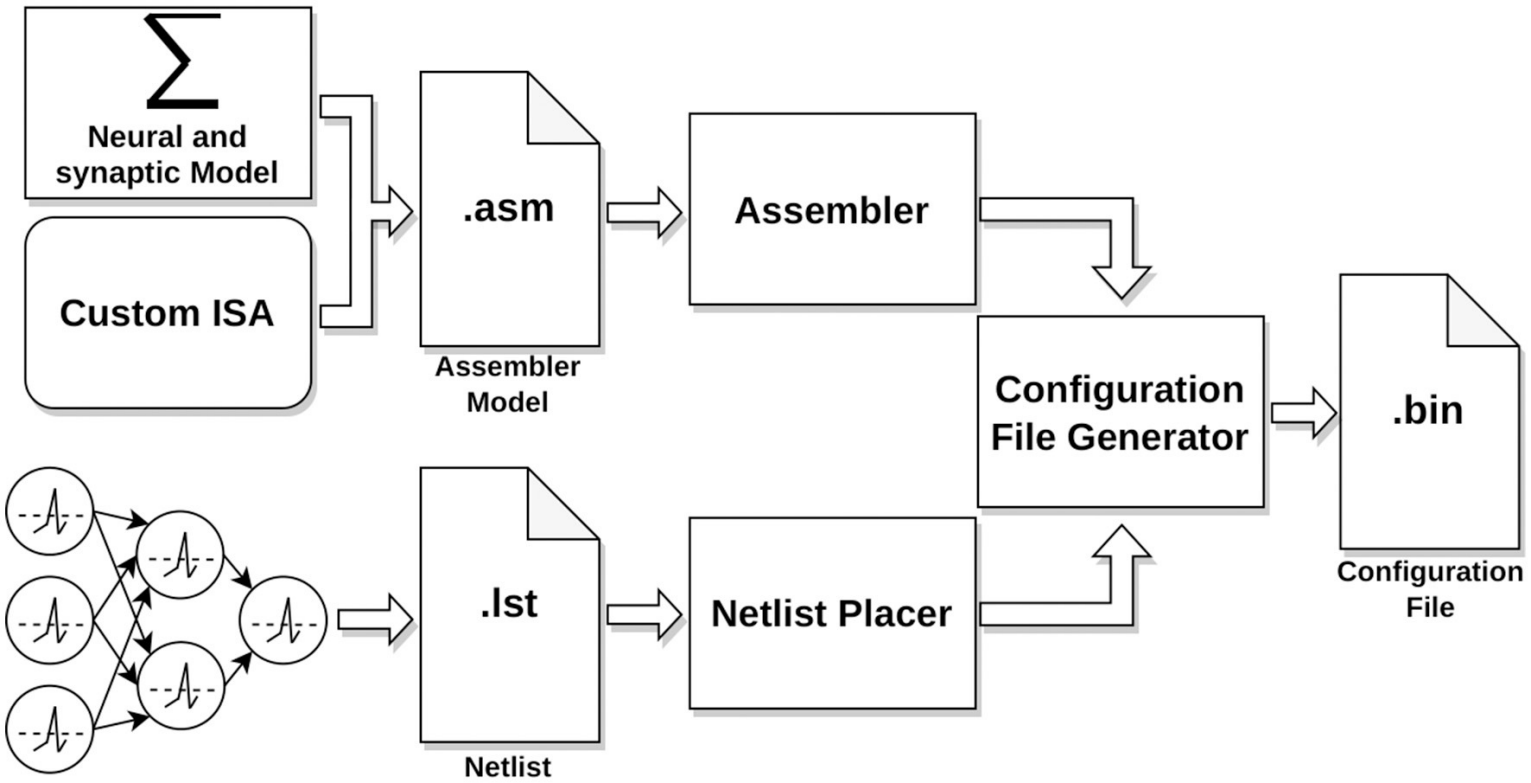
Workflow: neural modeling to configuration file generation.

The implementation process of a neural application in HEENS involves several stages as shown in Figure 4. Every application requires two files, the assembler model, that contains the neural and synaptic algorithms and the netlist. Both files are compiled, and with the use of custom-made Python tools, a configuration file is generated. This file is then transmitted from the PC to the HEENS hardware through the Ethernet interface during an initial configuration phase.

#### 2.5.1 Assembler model

The neural and synaptic model algorithms are encoded in a text file in assembly language based on the HEENS custom Instruction Set Architecture (ISA). The ISA has 64 defined instructions, optimized for working with spiking neural networks. HEENS operates with 16-bit precision integer arithmetic, which proves to be a good trade-off between accuracy and resources due to its programmability and multi-model capability. Lower-precision solutions are often limited to specific applications or need to be combined with higher resolution formats to achieve desired outcomes (Yun et al., 2023). On the other hand, using higher resolutions significantly increases resource usage, which may compromise the scalability and power efficiency of the solution (Das et al., 2018; Narang et al., 2018). It was experimentally found that the best suited resolution to operate is 10 μV per Least Significant Bit (LSB).

For the implementation of the model, Equations 1–13 were used, which describe soma dynamics, synaptic plasticity, and homeostasis. However, in order to support the long time constants required by homeostasis, a modification was made in Equation 12. Undersampling was implemented for its decay calculation, which involves calculating the exponential term only at certain number of steps instead of at every time step. Because of the very slow decay, the variation over short time intervals is so small that it cannot be detected by the architecture resolution. The constant used to define these time intervals between each decay is *t*_*spa*−*samp*_ with a value of 10 s.

**Figure 5:**
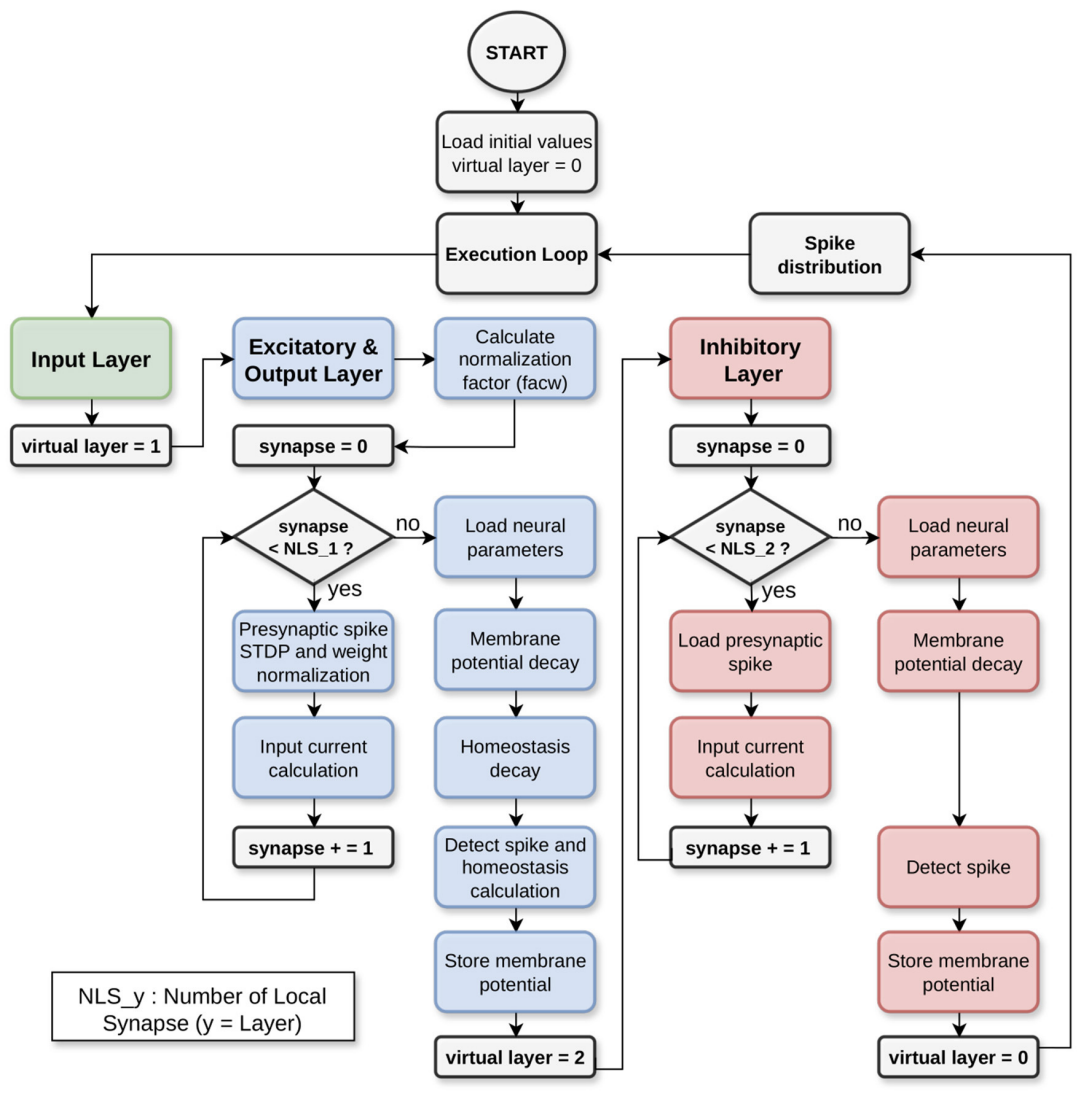
Workflow: assembler model.

The flowchart depicted in Figure 5 describes the model implementation considering the network structure. As described in Section 2.1, HEENS has the capability to time-multiplex each PE into different levels, called virtual layers. This allows the neurons between each layer to behave differently.

For the first layer (green), designated for input only, no action is taken other than increasing the layer counter. In the second layer (blue), excitatory and output neurons are implemented. Note that in addition to the LIF model algorithm, there are dedicated blocks to calculate plasticity and homeostasis; this layer is where training takes place.

The main algorithm consists of a loop of virtual layers. Blue boxes in Figure 5 correspond to the operations for the excitatory and output layers. Notice the synaptic loop and the and the neural operations for the current virtual layer. The last layer (red) is utilized for lateral inhibition, representing a condensed version of the preceding layer, focusing only on the implementation of the LIF model algorithm. It also calculates the synaptic loop and current virtual layer neuron.

This algorithm is also used during the testing stage, once the network has been trained. In Algorithm 1, the structure and format used to describe the model in pseudocode are shown. It is divided into three main parts: the definition of parameters and constants (see values in Table 1), the procedures outlined in Figure 5, and the main block that details the neuron and synapse execution and the spike distribution loop.

**Algorithm 1:**
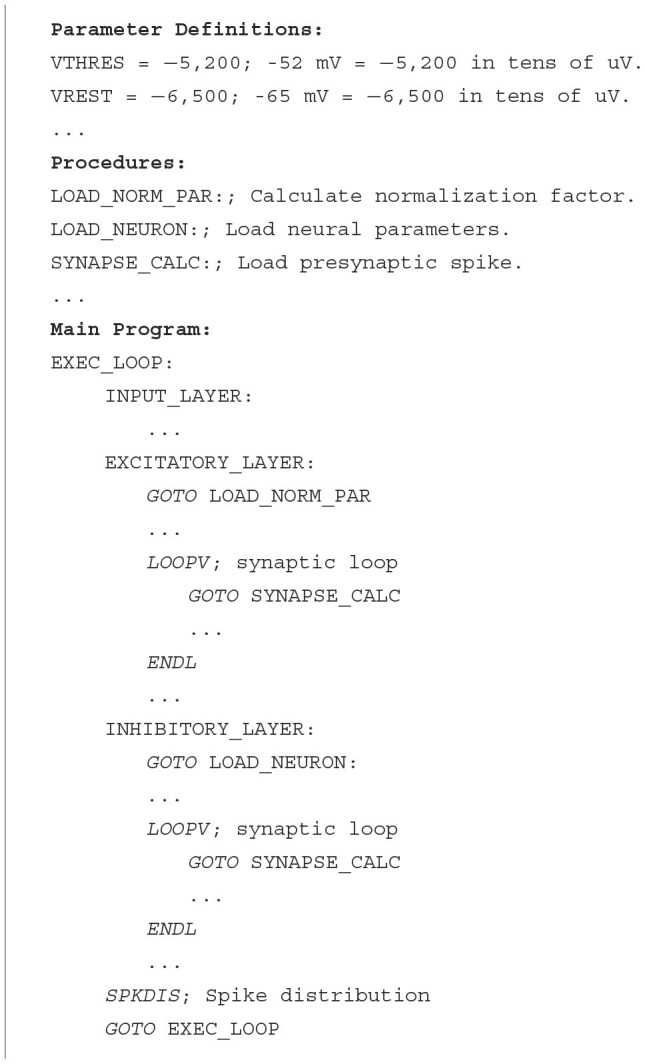
Pseudocode assembler Model MNIST application.

**Table 1:** LIF with STDP and homesotasis model parameters.

**Parameter**	**Value**	**Units**
**Soma parameters**		
Threshold voltage (*V*_*th*_)	−52	mV
Resting potential (*V*_*rest*_)	−65	mV
Synaptic potential difference (*k*)	−58	mV
Membrane decay time (τ_*m*_)	100	ms
**Synapse parameters**		
Current decay time (τ_*ex*_, τ_*ih*_)	1	ms
Synapse decay time (τ_*pre*_, τ_*post*_ )	20	ms
Increment factor presynapse (Δ_*pre*_)	0.01	
Increment factor postynapse (Δ_*post*_)	−0.01	
Target value excitatory layer (*W*_*norm*_)	20	
Maximum weigh excitatory synapse (*w*_*max*_)	1	
**Homeostasis parameters**		
Increment homesotasis (Δθ)	0.01	mV
Homeostasis decay time (τ_*hom*_)	1e6	ms
Spaced sampling time (*t*_*spa*−*samp*_)	10,000	ms
**Emulation parameters**		
Approach method	*Euler*	
Total Emulated neurons (*N*)	364	
LSB	10	μV
Time step (*dt*)	1	ms

#### 2.5.2 Netlist

The netlist provides a detailed description of the connections and parameters of the neural network, identifying both the source and the destination neuron, together with the associated neural and synaptic parameters (Algorithm 2).

**Algorithm 2:**
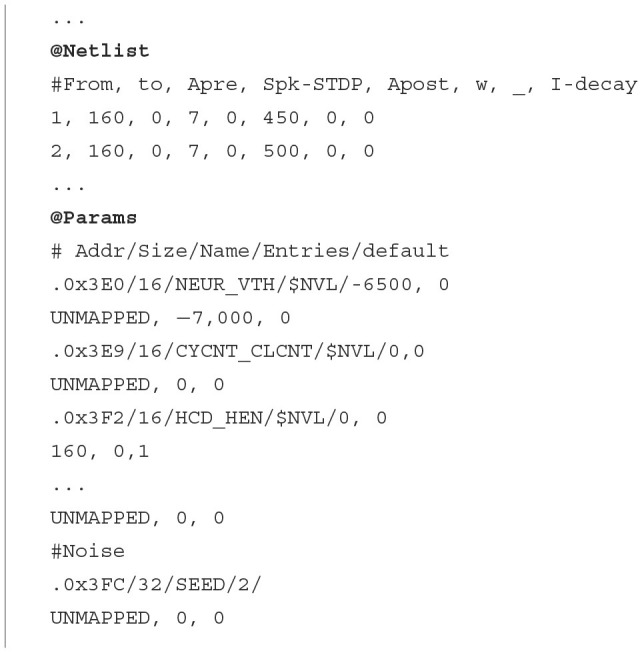
Netlist MNIST application.

As indicated in Section 2.1, each PE in HEENS contains two local memories shared among all its virtual layers. The Interconnection Memory is used to store the pre-synaptic connections of the PE neurons, while the SNRAM stores the neural and synaptic parameters.

Each connection defined in the netlist represents a synapse and it is stored in the Interconnection Memory according to the address of the pre-synaptic (source) neuron. In addition, depending on the synaptic model, multiple synaptic parameters can be associated with each synapse in the netlist file, the most important being the weight. These parameters are stored in the SNRAM, and the maximum number of definable parameters is limited by the memory size and the maximum number of synapses per neuron.

For this specific application, five memory spaces have been allocated, distributed among connection weights, parameters related to synaptic plasticity, and the exponential decay of synaptic current. The values for each synapse are initialized in the @*Netlist* section shown in Algorithm 2, where the first two values represent the identifiers of the source (pre-synaptic) and destination (post-synaptic) neurons of the synapse, followed by the synaptic parameters.

In addition to synaptic parameters that influence synaptic dynamics, neural parameters associated with the soma are also considered, being the most relevant the membrane potential. Other relevant parameters may vary depending on the model used. These parameters are defined and initialized at the end of the netlist file in the @*Params* section in Algorithm 2. Similarly to synapses, the number of neural parameters that can be defined is limited by the memory size.

For this particular case, eight memory locations per neuron have been allocated. These spaces are distributed among neural membrane potential storage, parameters required for weight and plasticity normalization, and those needed for homeostasis computation. In Algorithm 2, it can be observed that for each neural parameter, an initial memory address is established, along with default values depending on whether the neuron is mapped or not, as well as specific values as required by the model.

**Figure 6:**
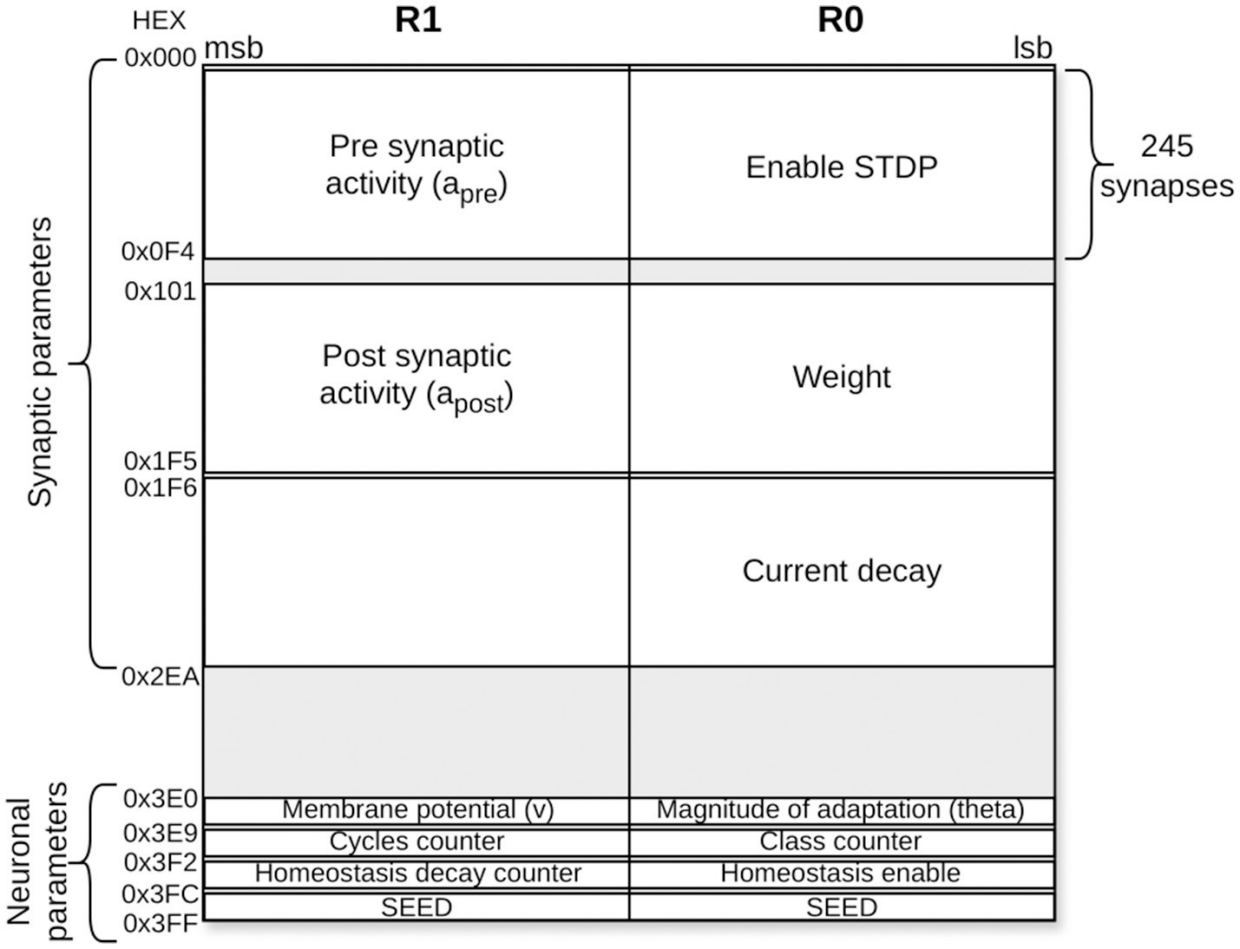
SNRAM memory mapping for an excitatory neuron in the second layer. Each synapse is allocated five memory spaces for synaptic parameters, while each neuron has eight memory spaces reserved for neural parameters.

Taking into account these synaptic and neural parameters, the memory map of an excitatory neuron is presented in Figure 6. The size of the SNRAM is 1024 × 32 bits. As seen in the Figure 6, each memory location consists of 32 bits, which are divided into two parts. The least significant 16 bits are associated with register R0, and the most significant 16 bits are associated with register R1. These two registers belong to the PE register bank, being both used within the assembler model algorithm described. In total, about 75% of the memory capacity is used for this application.

In the next section, the values listed in Table 1 are used to conduct the experiments. These values were chosen based on parameters used in previous research, such as the investigation reported by Diehl and Cook (2015). In addition, some parameters were adjusted through experimentation, testing, and tuning to better align the employed model with the specific characteristics of the experiments, and with the HEENS architecture. This approach ensured a parameter configuration tailored to the experiments, balancing consistency with previous work and adaptation to the specific needs of the study.

## 3 Experiments and results

### 3.1 Setup and real-time application execution

This section outlines the hardware and software requirements for preparing and executing the application in real-time. It involves the use of a PC, running a suite of Python-developed tools responsible for generating a configuration file, monitoring, and external input data transmission. The Xilinx Zynq ZC706 development board, which serves as the hardware's Master Chip, hosts the architecture implementation. To facilitate the transmission of external spikes without affecting HEENS operation, an Arduino Due was employed as a bridge between the PC and the Zynq. Lastly, the interface standards utilized include Ethernet for configuration and spike storage, HDMI for real-time monitoring, and USB for external input data transmission.

**Figure 7:**
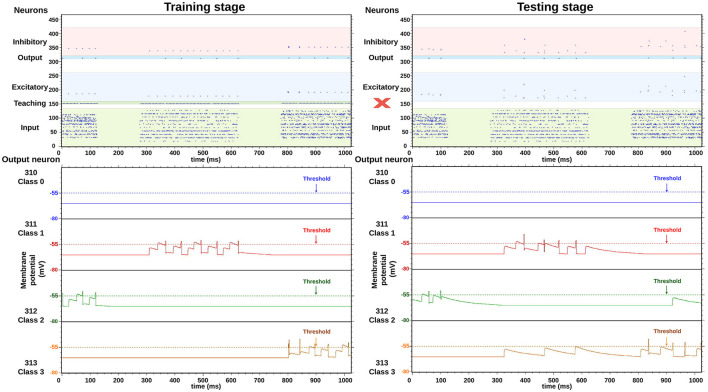
Real-time execution, comparison between training and testing stages, using the same input classes and the HEENS HDMI tool. The red X character is used to emphasize that during the testing phase, teaching inputs are disabled.

In Figure 7, two images obtained from the HEENS HDMI monitoring tool can be observed, one during the training phase and the other one in the testing stage. They depict the raster plot of neural activity within a 1,000 ms window. For didactic purposes, color bands have been included to group neurons according to their corresponding layers: green for the input layer, blue for the excitatory and output layer, and red for the inhibitory layer. The bottom part illustrates the membrane potential evolution of four selected output neurons. The differences between the two stages are as follows: in the first stage, the teaching signal is activated, leading to higher activity in the neuron corresponding to the trained class, while in the testing stage, the activity is lower but only reflects the network's response to the evaluated input. The same input data has been selected to clearly discern their differences.

### 3.2 Experimental results

In this section, the results of two experiments selected based on works reported by Iyer and Basu (2017) are presented. The approach follows the strategy of first evaluating a smaller dataset to explore and fine-tune the parameters used, as it involves less time and hardware resources. Once the values are determined, the second experiment is carried out to evaluate the complete dataset.

#### 3.2.1 5-class dataset experiment

**Figure 8:**
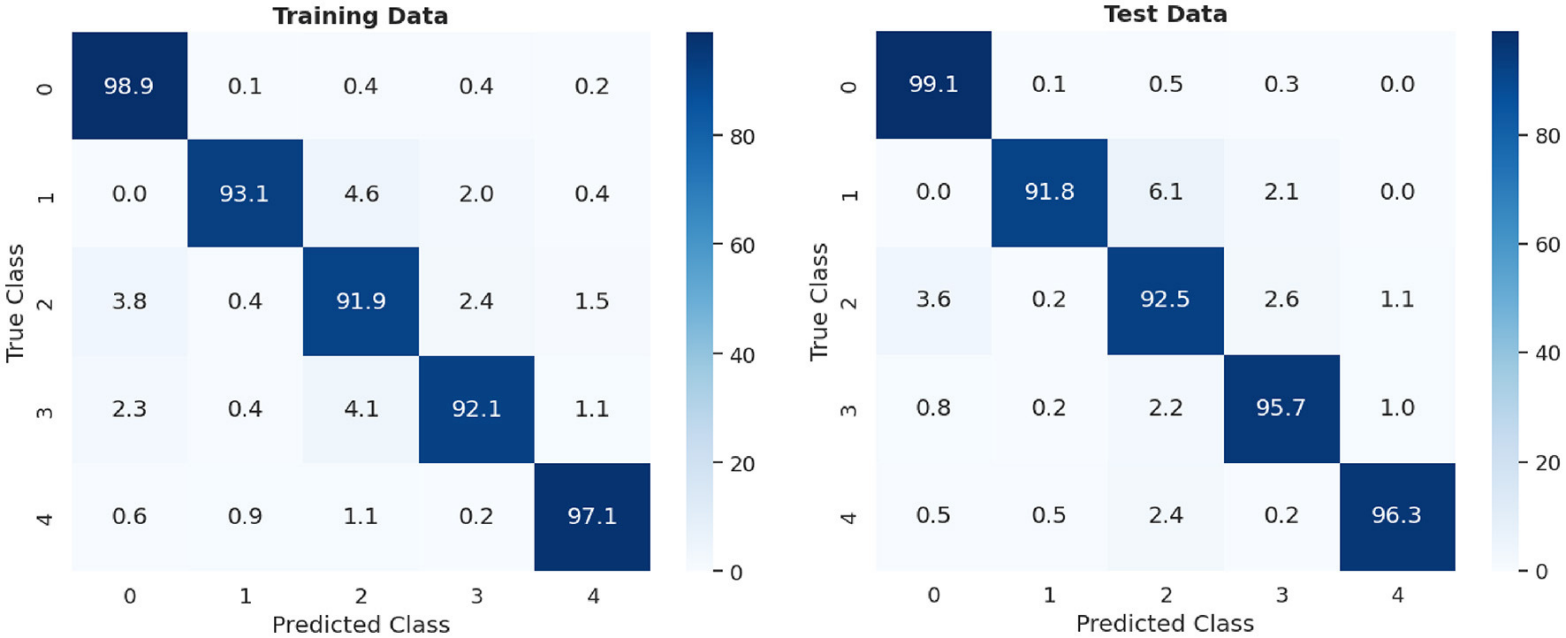
Confusion matrix of 10,000 MNIST digits: training vs. test data.

The digits chosen for the experiment were those that exhibited the greatest dissimilarity from each other (0, 1, 2, 3, and 4). The network configuration is identical to that outlined in Figure 3, with the only difference being that the groups of neurons per class in the excitatory layer consist of 20, as opposed to the original 10. For training, 15,000 different patterns were used, presented randomly over 2 epochs. For the evaluation stage, two sets of 5,000 different patterns each were used: the first set consisted of the training patterns themselves, and the second set corresponds to the test patterns. Figure 8, displays the confusion matrix of the conducted tests and the summary of the results are shown in Table 2.

**Table 2:** Classification accuracy 5-class experiment.

**Experiment**	**Dataset**	**Performance**
5-class	Training	94.62%
5-class	Test	95.08%

In Figure 8, the percentage of accuracy for each digit is shown for the training and test data scenarios, with *0* being classified with the highest precision and *2* with the lowest precision. Comparing the results shown in Table 2, it can be observed that the test data had a higher, but similar, accuracy percentage compared to the data used for training. This suggests an effective generalization of the model, indicating that it has learned representative patterns from the training data and is capable of applying them accurately to new unseen data.

#### 3.2.2 10-class dataset experiment

In this experiment, the classification of the 10 digits is evaluated. We chose to train with 15,000 classes out of the available 60,000 for 4 epochs. This decision was based on tests that determined that this range yielded the best results without overfitting the system, while also allowing for efficient management of execution time by reducing training times. Similarly to the previous case, for evaluation, two datasets are used: 10,000 patterns are selected from the training data and 10,000 from the test data. The classification results are summarized in Table 3.

**Table 3:** Classification accuracy 10-class experiment.

**Experiment**	**Dataset**	**Performance**
10-class	Training	82.85%
10-class	Test	82.22%

**Figure 9:**
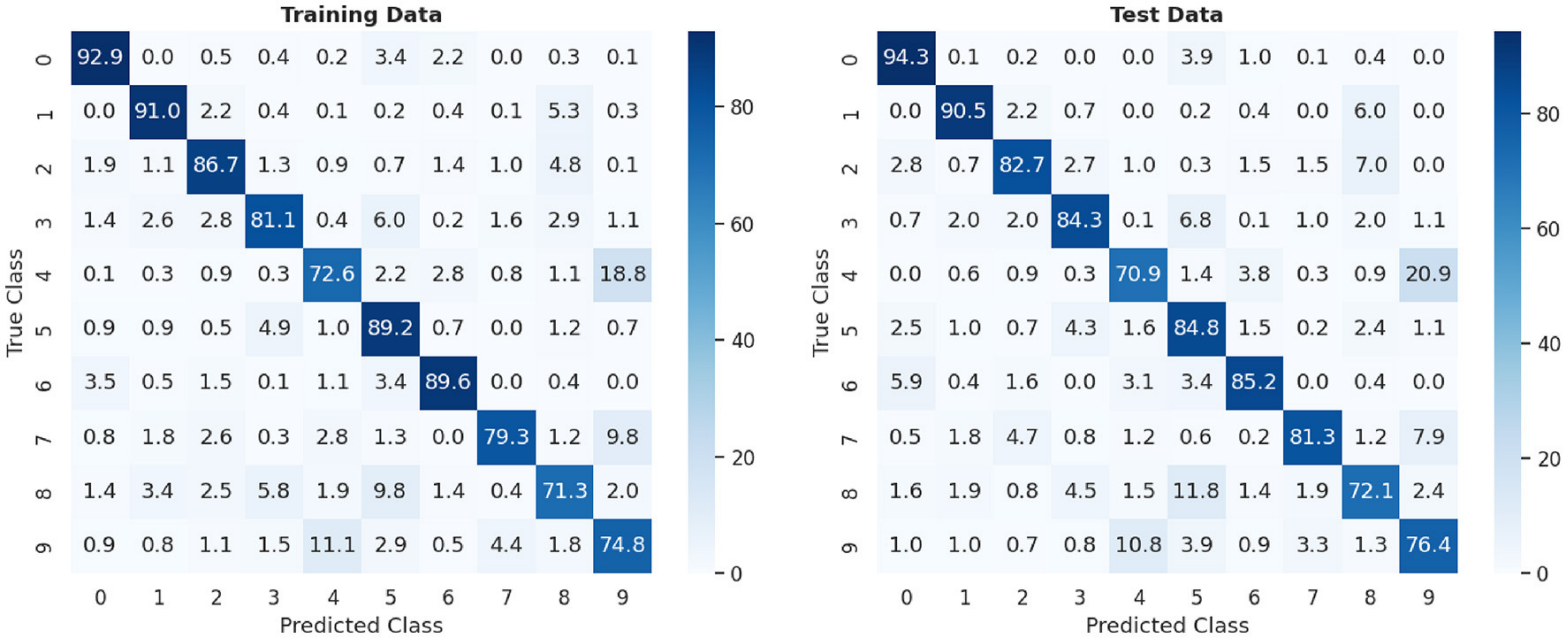
Confusion matrix of 20,000 MNIST digits: training vs. test data.

Figure 9 provides a detailed view of the confusion matrix generated during the experiment. It clearly illustrates how the recognition model has responded to each of the digits in the dataset. In particular, the precision of recognizing digits *0, 1, 2, 3, 5, 6* and *7* is particularly high, exceeding 80%, suggesting a remarkable ability to distinguish and classify these digits correctly. However, it should be noted that digits *4* and *9* exhibit lower precision, below 75%, as evidenced by the results shown in the confusion matrix. This lower precision may be attributed to the inherent challenges in distinguishing these digits, particularly when working with lower resolutions where their differences are less perceptible. Furthermore, digit *8* also shows a lower precision, as it is often confused with digit *5*. These results highlight potential areas for improvement in the model and suggest specific challenges in accurately classifying certain digits.

## 4 Discussion

### 4.1 Comparison with previous research

In current research, significant progress is being made in spiking neural networks and their applications in image classification, standing out for their low power consumption and high efficiency (Sadovsky et al., 2021). These advantages are particularly evident in neuromorphic architectures specifically designed for their implementation. However, challenges remain in terms of precision and development, preventing them from reaching the level of other alternatives, such as artificial neural networks (ANN). However, the ongoing exploration of novel models and network architectures is gradually narrowing this gap (Niu et al., 2023).

This section presents a comparison of the classification performance among different works based on the neural network architecture proposed by Diehl and Cook (2015), employing Spiking Neural Networks (SNNs) in the MNIST digit recognition task. The performance of each application is detailed in Table 4. It is worth noting that in all implementations, the results were evaluated considering the use of 100 neurons exclusively in the excitatory layer of the network, in accordance with the number of neurons implementable in a single-node HEENS prototype for practical reasons. The results of this comparison provide insight into how the proposed system compares to other existing applications.

**Table 4:** Comparison of classification performance.

**Network model**	**Input neurons**	**Resource**	**Supervision**	**Performance**
Diehl and Cook (2015)	28 × 28	Python & BRIAN	Unsupervised	82.00%
Querlioz et al. (2018)	28 × 28	C++ (Xnet)	Unsupervised	86.00%
Hao et al. (2020)	28 × 28	GENN	Supervised	83.67%
Guo et al. (2020)	28 × 28	Python & FPGA	Unsupervised	85.78%
Lee and Sim (2023)	28 × 28	Python	S1-U2	88.56%
**This work**	**12** **×** **12**	**HEENS**	**Supervised**	**82.22%**

Unsupervised: after training, each neuron is assigned a class “labeling”.

Supervised: labels are transmitted simultaneously with the training data.

S1-U2: indicates that the ratio of supervised and unsupervised learning used is 1:2.

GENN, GPU-enhanced neural networks.

When comparing the results of this study with others in terms of accuracy, we observe that while we surpass the results obtained by Diehl and Cook (2015), there are works that have superior results. However, it is important to note that each image has been down-scaled to 18% of its original size, resulting in a reduction of 640 neurons. Moreover, in contrast to all other studies, with the exception of Guo et al. (2020), who also used an FPGA but only implemented 25 physical neurons, the design tested in this study operates on a real-time architecture.

As indicated by the power consumption analyzer in Vivado (the Xilinx FPGA synthesis and implementation tool), the total On-Chip Power consumption is 4.39 W, derived from the sum of dynamic and static power components. It is important to highlight that the power estimation tool gives very approximate results, although it provides some first-order estimation. The dynamic power consumption is calculated at 4.13 W, which represents the power consumed by active components during operation. Additionally, there is a static power consumption of 0.25 W, representing the baseline power consumption of the FPGA even when no significant activity is occurring. This estimation of consumption is significantly less than the power consumption of a processor used in other solutions presented in Table 4, which typically ranges from 60 to 300 W (Prieto et al., 2022).

Table 5 compares several digital SNN hardware architectures with HEENS. While HEENS offers significant flexibility and programmability due to its multimodal and adaptable design, its power consumption is higher compared to other ASIC-based architectures. It is important to note that HEENS uses a Zynq platform instead of an ASIC, which typically results in higher power consumption but provides greater flexibility and reconfigurability, representing a significant advantage in the current stage of development. In contrast, the other architectures utilize ASIC technology, which is specifically optimized for low power consumption and high performance, albeit at the expense of flexibility.

**Table 5:** Digital SNN hardware architectures comparison.

**Hardware**	**Technology**	**Neurons—synapses core**	**Weight storage**	**On-chip learning**	**Time resolution**	**Neural model**	**Power consumption**
TrueNorth	ASIC
28 nm	256–64 k	1-bit	No	1ms	LIF	100 mW (per chip)
Spinnaker	ASIC
130 nm	1,000–1 M	16-bit	Yes	1 ms	Programmable	1 W (per chip)
Loihi2	ASIC
7 nm	8,192–937 k	8-bit	Yes	Variable^†^	Programmable	~1 W (per chip)
HEENS	Zynq FPGA
28 nm	1,280–41 k	16-bit	Yes	Variable^◇^	Multimodel &
Programmable	4.4 W^*^

^†^The time resolution corresponds to the algorithmic time and is unrelated to real-time. ^♢^HEENS can be configured with time steps from 2.5 μs to 16 ms (application-dependent). Default: 1 ms. ^*^From Vivado Integrated Design Environment power consumption report.

### 4.2 Implications and limitations

The HEENS architecture represents a promising alternative for modeling SNNs and their applications, due to its flexibility in implementing programmable neural and synaptic models with low resource and power overhead. This flexibility stands out in the realm of neuromorphic architectures, where many of them are designed around specific models, requiring a complete restructuring for any modification (Benjamin et al., 2021).

The use of dedicated memory per PE, capable of mapping different parameter types, facilitates the development of models with many levels of complexity. However, it is important to acknowledge that increased algorithm complexity implies higher hardware resource consumption, especially in the specific case of the FPGA. This poses a current limitation when implementing the application with the amount of original neurons presented in works such as Diehl and Cook (2015), which hover around 1,000 neurons in total with about 900 synapses per neuron in the case of the neuron with maximum interconnection.

Therefore, based on the number of available PEs and synapses, the reduction of the input image size was considered, at the cost of a loss in classification precision. Consequently, the implemented application uses 364 neurons with 245 synapses in the case of the neuron with the highest interconnectivity. It is important to note that while working with lower-resolution images has the advantage of lower computational cost, the loss of detail impacts precision and complicates the extraction of important features for classification.

**Figure 10:**
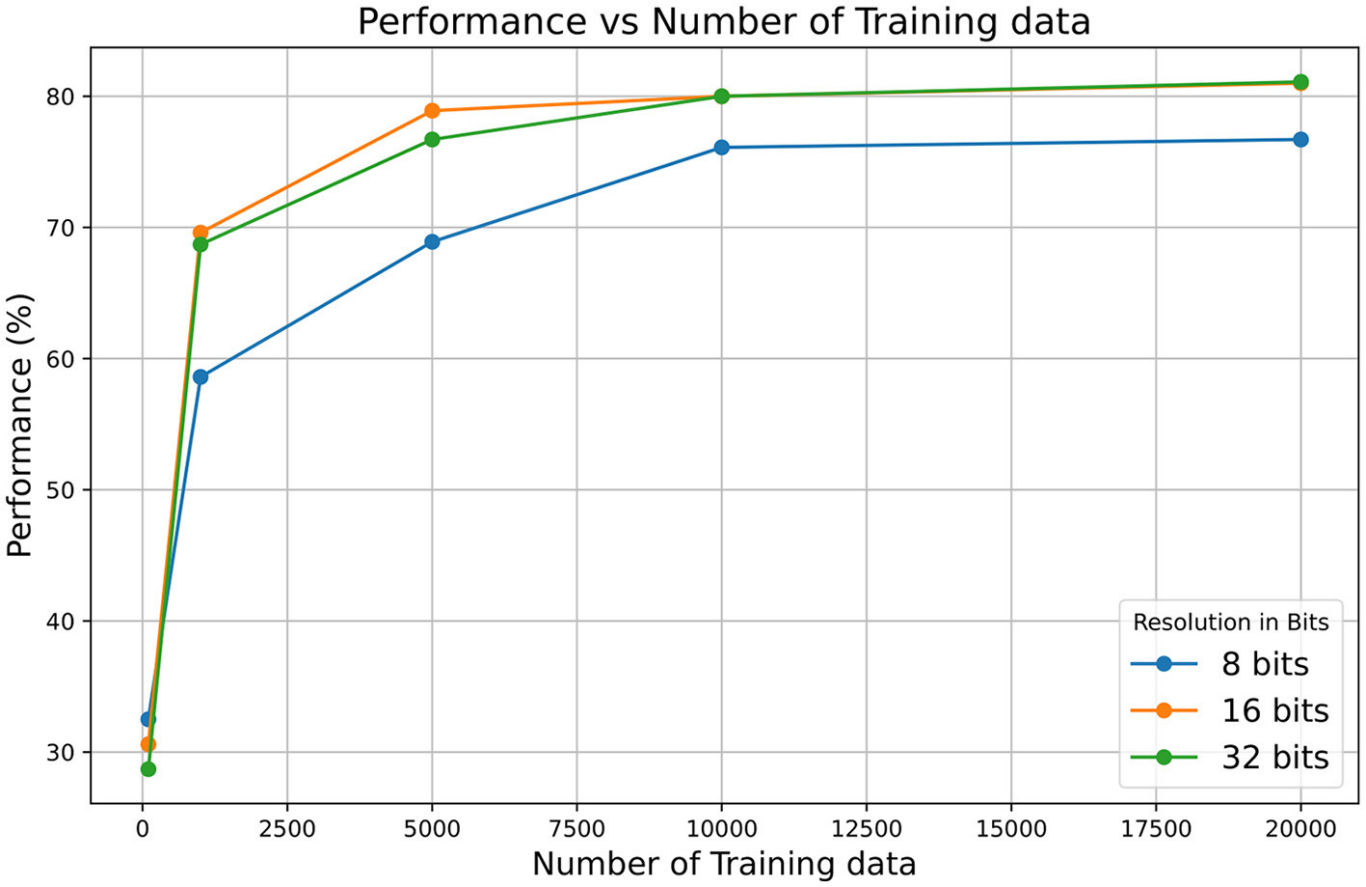
Performance vs. number of training data for different resolutions.

In addition, it should be noted that the architecture uses 16-bit integer arithmetic, which may potentially impact the calculation precision. Tests have been conducted using MATLAB to simulate the model's behavior and its response to different precisions (8, 16, and 32 bits) applied to the membrane potential variable and the synaptic input current. It was found that using other variables at 8 bits did not provide sufficient resolution. The results are shown in Figure 10, where it can be observed that the 16-bit resolution achieves higher performance compared to the 8-bit resolution and matches the 32-bit resolution performance for long training. However, using higher resolutions, such as 32-bit, significantly increases resource usage, including memory and power consumption, which may compromise the solution and its scalability. For instance, increasing the resolution of neuronal and synaptic parameters from 16 to 32 bits implies a substantial increase in the size of the SNRAM memory (Figure 6). The FPGA used consists of 545 BRAM blocks of 36 kb, of which 60.55% are currently used. After the change to 32 bits, the utilization would increase to 89.9%. This increase represents a significant impact on resource usage and also affects power consumption, as BRAM blocks are among the highest consumers, accounting for 32% of the total power consumption (data obtained from the VIVADO Synthesis tool).

To maximize the performance of the numeric formats, as indicated before, a Least Significant Bit (LSB) of 10 μV is adopted, and all values are scaled accordingly. This meticulous approach ensures that the system maintains accuracy and reliability despite inherent hardware resource limitations and numerical representation challenges.

### 4.3 Future directions

Although the current HEENS implementation exhibits limitations in terms of hardware resources, it is important to note that efforts are being made to overcome these constraints. Currently, work is underway on implementing a hierarchical system and developing an Application-Specific Integrated Circuit (ASIC), that promises to maximize performance. In this regard, a prototype is being developed using a cost-effective 28 nm CMOS process. According to the PE physical layout obtained in the 28 nm TSMC technology, it is estimated that a ring of 10 nodes of 16 × 16 PE multiprocessors can be integrated in a 1 cm^2^ chip. Using a much more aggressive technology scaling, using a 3 nm technology, it would become feasible to integrate 640 nodes in a hierarchy of six rings of about 100 nodes each. This would be a network of 1.3 million neurons in a single chip.

To support the increase of PEs beyond the previously described ring limitation of 127 nodes, it is crucial to improve their level of interconnection. To address this aspect, a hierarchical structure inspired by the modularity of the brain and its hierarchical configuration of densely connected nodes is being developed (Friston, 2008; Akiki and Abdallah, 2019). Efforts are underway to extend this solution to higher hierarchical levels of rings, which combined with the ASIC will provide a comprehensive solution for the development and evaluation of the system in large-scale applications. This includes evaluating the system's performance using larger datasets such as CIFAR-10 and CIFAR-100 (Krizhevsky, 2009) to ensure robust and scalable results. This strategic approach to improving the implementation demonstrates a continued commitment to optimizing its performance and adaptability to the demands of specific applications in the field of spiking neural networks.

The HEENS architecture offers promising insights for future research and development in the field of neuromorphic computing providing a platform for exploring novel algorithms and network architectures. Specifically, in the field of neuroscience thanks to its bioinspired organization. Currently, efforts are underway to replicate the behaviors observed in *in vitro* neuronal cultures, as documented in the works of Orlandi et al. (2013) and Faci-Lázaro et al. (2019). This opens up opportunities to detect and to address existing hardware limitations, such as the trade-off between algorithm complexity and hardware resource consumption.

Furthermore, as researchers continue to push the boundaries of SNNs, HEENS could serve as a valuable tool for studying the principles of neural computation and for developing more efficient and adaptable systems. Using its capabilities, future advancements in HEENS could lead to breakthroughs in areas ranging from pattern recognition to cognitive computing, ultimately advancing the frontier of neuromorphic computation.

## 5 Conclusion

In the broader context of SNNs and their application in image classification, our study contributes to the ongoing discourse surrounding the balance between accuracy and efficiency. Our achieved accuracy of 82.22% is not far from other published SNN results and it is crucial to emphasize the trade-offs inherent in our approach. Using a scaled down input size and leveraging a real-time architecture, we prioritize efficiency without sacrificing significantly on performance. This approach aligns with the growing emphasis on low-power computing and edge computing applications, where energy efficiency is paramount. Furthermore, our findings highlight the adaptability of SNNs in real-world scenarios, particularly in tasks where real-time processing and low power consumption are critical considerations. Moving forward, further research could focus on refining our methodology to improve accuracy while maintaining efficiency, ultimately advancing the practical applications of neuromorphic computing in various domains. Furthermore, the proposed approach can be very helpful in pushing forward the development of sustainable and efficient machine learning.

Summarizing, the proposed neuromorphic hardware provides a sweet tradeoff between flexibility, performance (resources and power) and scalability. In this work, the theoretical idea from the hardware architecture proposal has been demonstrated by proving the execution capability of HEENS implemented on an FPGA platform with the MNIST dataset.

## Data availability statement

Publicly available datasets were analyzed in this study. This data can be found here: http://yann.lecun.com/exdb/mnist/.

## Author contributions

BV-M: Writing – original draft, Writing – review & editing, Conceptualization, Formal analysis, Investigation, Methodology, Supervision, Data curation, Software. JM: Conceptualization, Formal analysis, Investigation, Methodology, Supervision, Writing – review & editing, Writing – original draft, Funding acquisition, Project administration. MZ: Conceptualization, Formal analysis, Investigation, Methodology, Supervision, Writing – review & editing, Writing – original draft.
